# *Tlr2* deficiency does not limit the development of left ventricular hypertrophy in a model of transverse aortic constriction induced pressure overload

**DOI:** 10.1186/s12952-016-0050-3

**Published:** 2016-04-25

**Authors:** Tippaporn Bualeong, Sied Kebir, Dorothea Hof, Lina Goelz, Mathias Graewe, Stefan Felix Ehrentraut, Pascal Knuefermann, Georg Baumgarten, Rainer Meyer, Heidi Ehrentraut

**Affiliations:** Physiology Department, Medical Science Faculty, Naresuan University, Phitsanulok, 65000 Thailand; Institute of Physiology II, University Hospital Bonn, Nussallee 11, 53115 Bonn, Germany; Department of Neurology, University Hospital Bonn, Sigmund-Freud-Str. 25, 53127 Bonn, Germany; Polyclinic of Orthodontics, University of Bonn, Welschnonnenstraße 17, 53111 Bonn, Germany; Department of Anaesthesiology and Intensive Care Medicine, University Hospital Bonn, Sigmund-Freud-Straße 25, 53127 Bonn, Germany

**Keywords:** TLR2, Innate immunity, Aortic constriction, Cardiac hypertrophy

## Abstract

**Background:**

Toll-like receptors (TLRs) are involved in a variety of cardiovascular disorders, including septic cardiomyopathy, ischemia/reperfusion, heart failure, and cardiac hypertrophy. Previous research revealed that TLR4 promotes cardiac hypertrophy in vivo. Therefore, we investigated whether TLR2 is also involved in the development of cardiac hypertrophy.

**Methods:**

*Tlr2* deficient and wild type mice were subjected to transverse aortic constriction (TAC) or sham operation procedure. Left ventricular, heart and lung weights as well as hemodynamic parameters were determined after 3, 14 or 28 days. Real-time RT PCR was used to evaluate left ventricular gene expression. Protein content was determined via ELISA.

**Results:**

TAC increased systolic left ventricular pressure, contraction and relaxations velocities as well as the heart weight in both genotypes. *Tlr2* deficiency significantly enhanced cardiac hypertrophy after 14 and 28 days of TAC. Left ventricular end-diastolic pressure and heart rate increased in *Tlr2*^*−/−*^ TAC mice only. Fourteen days of TAC led to a significant elevation of ANP, BNP, TGFβ and TLR4 mRNA levels in *Tlr2*^*−/−*^ left ventricular tissue.

**Conclusion:**

These data suggest that *Tlr2* deficiency may promote the development of cardiac hypertrophy and ventricular remodeling after transverse aortic constriction.

## Background

Toll-like receptors (TLRs) are involved in a variety of cardiovascular disorders, including myocardial dysfunction during sepsis, ischemia/reperfusion, heart failure, cardiac hypertrophy, and atherosclerosis. Previous research revealed that TLR4 promotes cardiac hypertrophy in vivo [1, 2], and that the endogenous TLR4 ligand fibrinogen induces a hypertrophic response of cardiomyocytes [3]. Like *Tlr4*^−/−^ mice, *Tlr2*^*−/−*^ and *Tlr9*^*−/−*^ mice responded to myocardial infarction with reduced injury [4–7].

Endogenous ligands such as heat shock proteins HSP60, HSP70, and HSP96, HMGB1, biglycan, and β-defensin, have been shown to activate NF-κB via TLR2 and TLR4 in non-cardiac cells. Cardiac overload increased HSP70 and HSP72 expression in myocardium [8, 9] and targeted over-expression of HSP56 promoted hypertrophy of cultured cardiac muscle cells [10]. Overall, these studies suggest a strong correlation between TLR signaling and heart disease. We aimed to clarify, whether TLR2 contributes to the development of cardiac hypertrophy. Therefore, we investigated the influence of TLR2 deficiency on transverse aortic constriction (TAC) induced pressure overload for up to 28 days.

## Results

### TLR2-deficiency increases cardiac hypertrophy after transverse aortic constriction

**Fig. 1:**
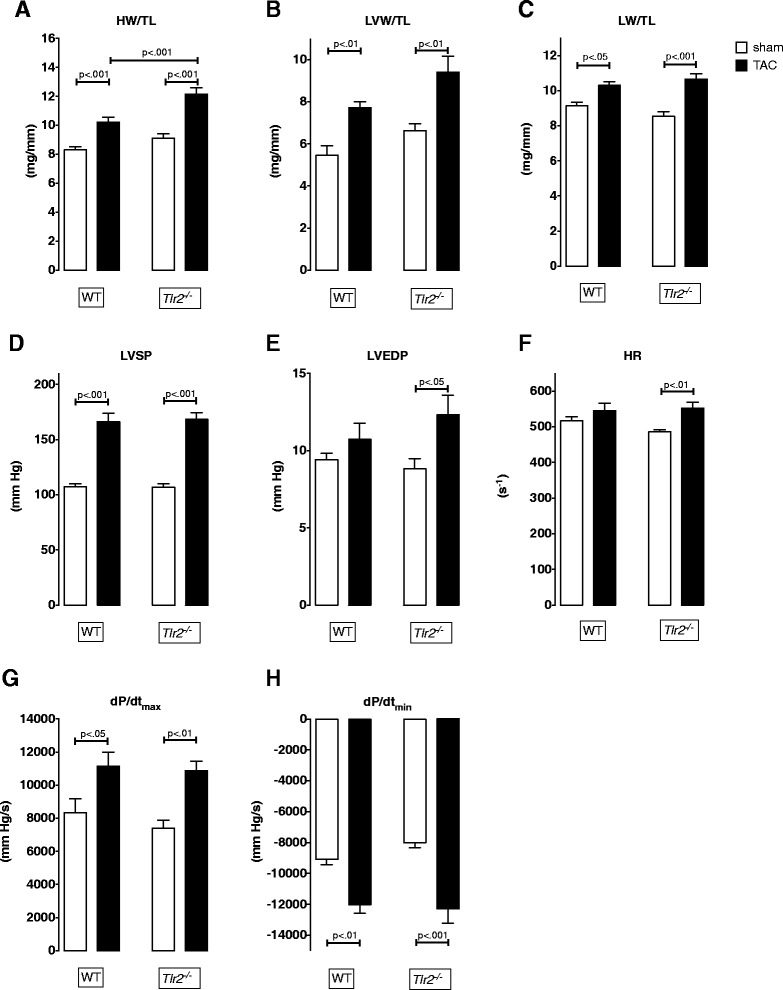
Fourteen days of aortic constriction differentially modify biometric and hemodynamic measurements in male *Tlr2* ^*+/+*^ and *Tlr2* ^*−/−*^ mice. **a**-**c** Heart weight (HW), left ventricular (LVW) and lung weight (LW) were normalized to tibia lengths (TL). Ratios increased in all TAC groups in comparison to sham groups. *Tlr2* ^*−/−*^ mice developed a significantly elevated HW/TL ratio compared to *Tlr2* ^*+/+*^ mice (mean ± SEM, *n* = 5–11/group). **d**-**h** TAC altered left ventricular systolic pressure (LVSP), contraction (dP/dt_max_) and relaxation (dP/dt_min_) velocity in both genotypes, but changed left ventricular end-diastolic pressure (LVEDP) and heart rate (HR) in *Tlr2* ^*−/−*^ mice only (mean ± SEM, One-way ANOVA with Sidak post-hoc testing, *n* = 9–12/group)

Age and weight matched WT or *Tlr2*^*−/−*^ male mice displayed a significant increase of heart (HW) and left ventricular weight (LVW) 14 days after TAC surgery (Fig. 1a, b). Normalization of LVW to tibia length (TL) confirmed that transverse aortic constriction accounted for LVW differences between TAC and sham groups. We also observed a significant increase of lung weight (LW)/TL ratio in both TAC groups compared to the respective sham group (Fig. 1c). The extent of cardiac hypertrophy was increased in *Tlr2*^*−/−*^ versus *Tlr2*^*+/+*^ mice as demonstrated by a 22.1 % higher HW/TL ratio (*p* < 0.01) and 19.2 % elevated LVW/TL ratio (not significant).

TAC induced a significant elevation of left ventricular systolic pressure (LVSP) in both genotypes (*p* < 0.001; Fig. 1d). Overall, left ventricular end-diastolic pressure (LVEDP, Fig. 1e) and heart rate (HR; Fig. 1f) were elevated in the two TAC groups but reached the level of significance only in *Tlr2*^*-/ -*^ mice (*p* < 0.05). Aortic constriction increased contraction (dP/dt _max_; Fig. 1g) and relaxation velocity (dP/dt _min_; Fig. 1h) in both genotypes.

### mRNA expression of hypertrophy related genes is enhanced in Tlr2^−/−^ mice

**Fig. 2:**
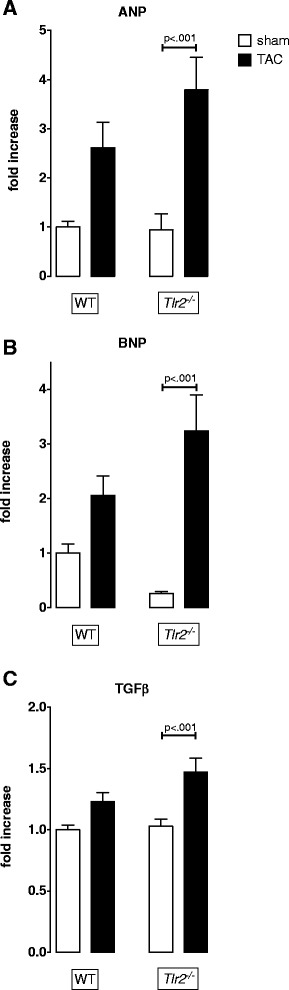
Measurement of pro-hypertrophic mediator mRNA expression as observed 14 days after aortic constriction by quantitative real-time PCR. Atrial natriuretic peptide (ANP) (**a**), B-type natriuretic peptide (BNP) (**b**), and transforming growth factor (TGF)-β (**c**) were increased in *Tlr2* ^*−/−*^ mice post TAC (mean ± SEM, One-way ANOVA with Sidak post-hoc testing, *n* = 8/group)

Since we monitored an influence of TLR2 signaling on cardiac measures, we analyzed whether the hypertrophy related genes atrial natriuretic peptide (ANP), b-type natriuretic peptide (BNP) and transforming growth factor (TGF)β reflect these findings (Fig. 2 a-c). Fourteen days of pressure overload induced a significant up-regulation of natriuretic peptides ANP and BNP (*p* < 0.001) as well as pro-hypertrophic TGFβ (*p* < 0.01) in *Tlr2*^*−/−*^ mice but not in wild type mice.

### Twenty-eight days of TAC do not further impair cardiac hypertrophic and hemodynamic function in Tlr2^+/+^or Tlr2^−/−^ mice

**Fig. 3:**
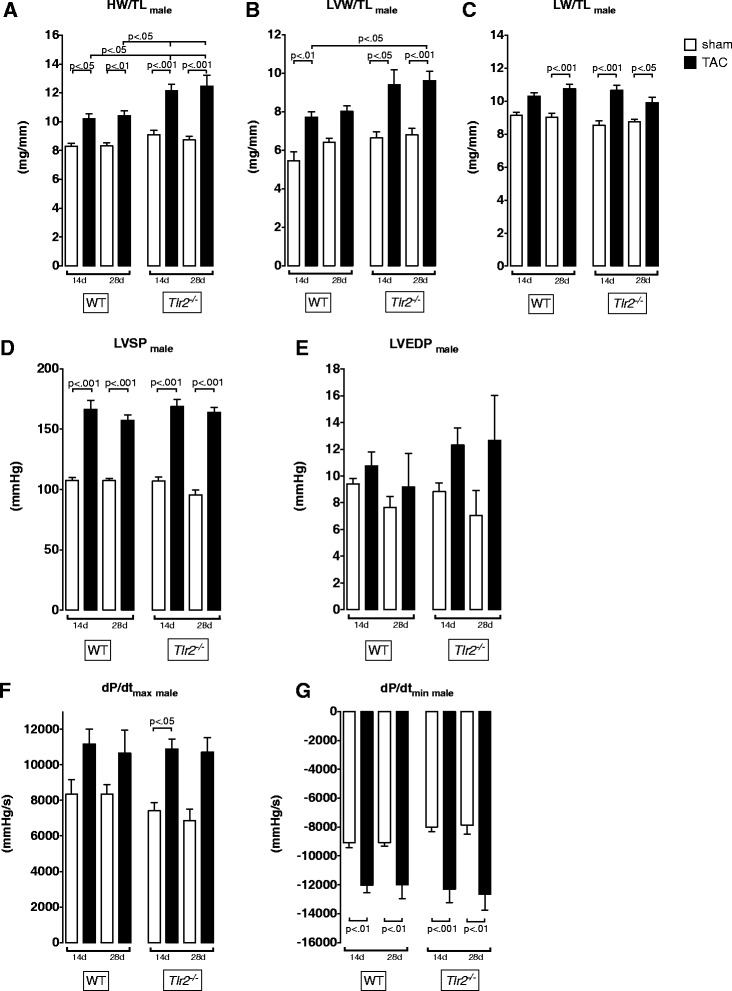
Comparative analysis of biometric and hemodynamic differences after 14 and 28 days of aortic constriction in male WT and *Tlr2* ^*−/−*^ mice. An extended duration of TAC had no additional impact on biometric measures (**a**-**c**) and hemodynamic function (**d**-**g**) and did not cause accelerated decompensation in any group (mean ± SEM, One-way ANOVA with Tukey post-hoc testing, **a** *n* = 7–11/group, **b** *n* = 6–13/group)

Since we observed differences in biometric parameters and hypertrophy related genes after 14 days, we assumed that the increased cardiac hypertrophy in *Tlr2*^*−/−*^ mice might lead to a decompensated heart failure with impaired cardiac function over time. Therefore, we extended the duration of aortic constriction to 28 days and repeated biometric and hemodynamic measurements (Fig. 3). In *Tlr2*^*−/−*^ mice, HW/TL and LVW/TL remained significantly elevated compared to *Tlr2*^*+/+*^ (*p* < 0.05) mice (Fig. 3 a+b). Hemodynamic function was not further impaired after 4 weeks and we detected no differences between the two genotypes (Fig. 3 d-g).

### The TLR2 effect on cardiac hypertrophy development is gender independent

Previous studies revealed that gender modifies the response to cardiac overload [11]. Therefore, we tested whether we also observe a gender dependent interaction between TLR signaling and LV remodeling.

**Fig. 4:**
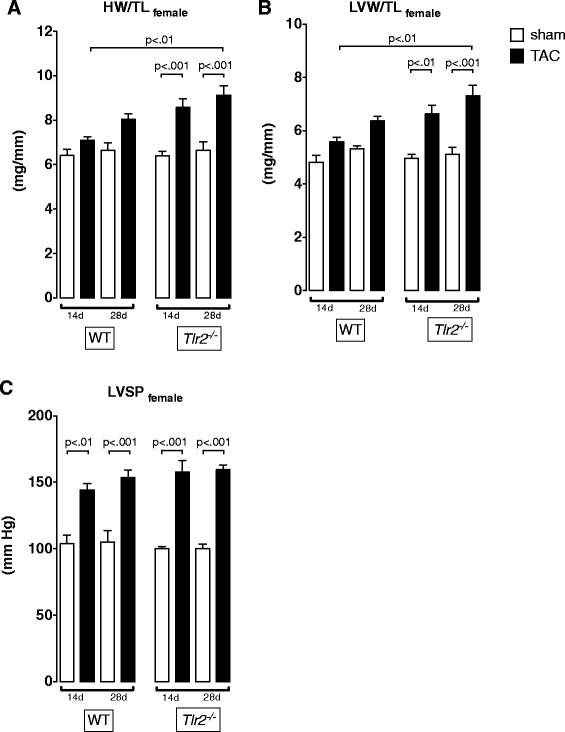
Increased hypertrophy development in *Tlr2* ^*−/−*^ mice was not gender specific. Comparative analysis of biometric (**a**, **b**) and hemodynamic differences (**c**) after 14 and 28 days of aortic constriction in female *Tlr2* ^*+/+*^ and *Tlr2* ^*−/−*^ mice demonstrated, that *Tlr2* ^*−/−*^ females also developed more pronounced hypertrophy than WT mice (mean ± SEM, One-way ANOVA with Tukey post-hoc testing, *n* = 7–11/group)

We repeated 14 and 28 days of aortic constriction in female mice. Cardiac hypertrophy was less prominent in female mice. After TAC, HW/TL as well as LVW/TL ratios were significantly increased in *Tlr2*^*−/−*^ but not *Tlr2*^*+/+*^ mice (Fig. 4 a+b). However, LVSP was significantly elevated in both genotypes after TAC (Fig. 4 c). Alterations in LVEDP were not detectable (data not shown).

### Differential regulation of extracellular matrix related genes after 14 days of TAC

In a previous publication by Mersmann et al. the authors reported left ventricular dilation, pronounced matrix remodeling characterized by reduced collagen and decorin density in the infarct scar of *Tlr2*^*−/−*^ mice 28 days after myocardial ischemia/reperfusion injury [12]. We assumed that alterations in extracellular matrix (ECM) composition might also explain the observed differences in cardiac hypertrophy. Therefore, we measured the expression of ECM components as well as ECM degrading enzymes in our samples (Table 1).

**Table 1:** mRNA expression profiles of extracellular matrix related genes 14 days after aortic constriction in male wild type and *Tlr2* deficient mice

	WT sham	WT TAC	Tlr2^−/−^ sham	Tlr2^−/−^ TAC
Col1α1	1.00 ± 0.18	2.91 ± 0.43^a^	4.06 ± 0.67^b^	2.44 ± 0.35
Col3α1	1.00 ± 0.19	3.46 ± 0.46^a^	0.91 ± 0.10	2.52 ± 0.30^c^
Col4α1	1.00 ± 0.07	1.74 ± 0.21	1.26 ± 0.07	2.62 ± 0.44^c^
Biglycan	1.00 ± 0.06	1.91 ± 0.19^a^	0.88 ± 0.11	1.82 ± 0.18^c^
Decorin	1.00 ± 0.04	1.12 ± 0.06	0.90 ± 0.08	1.11 ± 0.09
Elastin	1.00 ± 0.32	2.94 ± 0.41^a^	0.69 ± 0.09	1.81 ± 0.22^c,d^
LOX	1.00 ± 0.25	2.96 ± 0.46^a^	0.84 ± 0.10	2.20 ± 0.37^c^
MMP2	1.00 ± 0.12	2.08 ± 0.36^a^	1.40 ± 0.19	1.73 ± 0.25
MMP9	1.00 ± 0.13	1.12 ± 0.15	1.08 ± 0.14	1.41 ± 0.17
MMP13	1.00 ± 0.09	1.68 ± 0.20^a^	1.42 ± 0.15	1.75 ± 0.17

Values are normalized to wild type sham group, mean ± SEM, *n* = 8/group. Significant differences (*p* < 0.05, One-way ANOVA with Sidak post-hoc testing) between relevant groups are indicated by identical superscripted letters

^a^WT sham vs. WT TAC

^b^WT sham vs. Tlr2−/−

^c^Tlr2^−/−^sham vs. Tlr2^−/−^ TAC

^d^WT TAC vs. Tlr2^−/−^ TAC

After TAC we detected no major differences between genotypes in the expressions of pro-collagen type 1α 1 (COL1α1), COL3α1 and COL4α1 mRNA. Interestingly, COL1α1 showed a significant fourfold increase in *Tlr2*^*−/−*^ mice at baseline. We detected an elevation of the proteoglycan biglycan (*p* < 0.05) and weak decorin induction (not significant) in both genotypes after TAC. We observed a significant increase in TAC induced mRNA expression of the structural protein elastin in both genotypes. Wild type cardiac tissue elastin levels were higher than those measured in *Tlr2*^*−/−*^ hearts (*p* < 0.05). Lysyl oxidase (LOX) catalyzes the cross-linking between collagens and elastins. LOX mRNA expression was elevated by TAC in both genotypes (*p* < 0.05).

Matrix metalloproteinases (MMPs) regulate extracellular matrix degradation and synthesis, thereby controlling cardiac remodeling [13]. MMP2 and −13 but not MMP9 mRNA expression increased significantly (*p* < 0.05) in wild type mice after TAC.

### Modulation of TLR1 and −4 mRNA expression due to 14 days of aortic constriction

Three days of cardiac pressure overload have been shown to modulate the expression of TLRs [14]. Release and recognition of endogenous TLR ligands might modulate the expression of their respective receptors, and differentially alter the sensitivity towards the respective ligands in *Tlr2*^*−/−*^ deficient mice. Thus, we determined CD14, TLR-1, −2, −4, −6 and −9 mRNA after 14 days of TAC (Table 2). TLR1 mRNA expression was significantly upregulated in both genotypes after 14 days of aortic constriction (*p* < 0.05). Upregulation of TLR4 mRNA expression reached the level of significance in *Tlr2*^*−/−*^ mice (*p* < 0.05). Alteration of other pattern recognition receptors past TAC was only moderately induced.

**Table 2:** Pattern recognition receptor mRNA expression profile 14 days after aortic constriction in male wild type and *Tlr2* deficient mice

	WT sham	WT TAC	Tlr2^−/−^ sham	Tlr2^−/−^ TAC
TLR1	1.00 ± 0.12	1.92 ± 0.25*	0.92 ± 0.19	1.68 ± 0.10*
TLR4	1.00 ± 0.06	1.31 ± 0.11	1.05 ± 0.07	1.59 ± 0.16*
TLR6	1.00 ± 0.09	1.28 ± 0.12	1.28 ± 0.13	1.50 ± 0.08
TLR9	1.00 ± 0.09	1.54 ± 0.22	1.07 ± 0.07	1.55 ± 0.10
CD14	1.00 ± 0.08	1.49 ± 0.19	0.98 ± 0.19	1.57 ± 0.23

Values are normalized to wild type sham group, mean ± SEM, *n* = 8/group

*indicates significant differences to respective sham group (*p* < 0.05, One-way ANOVA with Sidak post-hoc testing)

### Mediator and PRR expression 3 days after TAC

We assumed that TLR4 induction observed in *Tlr2*^*−/−*^ mice after 14 days of TAC may reflect a dysregulated and prolonged upregulation of TLR4*,* thereby increasing endogenous TLR4 ligand binding and enhancing the pro-inflammatory cytokine response [1, 2, 15–18]. Therefore, we examined pattern recognition receptor (PRR) as well as pro-inflammatory cytokine regulation after 3 days of pressure overload. However, we observed no major differences in PRR induction between the groups (Table 3). Overall, pro-inflammatory cytokine mRNA expression increased TAC dependent. However, only IL-6 levels were significantly increased in *Tlr2*^*−/−*^ TAC mice. Both TAC groups displayed an elevation of IL-6 protein. However, none of these alterations were significant at this time point (Table 4).

**Table 3:** mRNA expression profile of PRRs 3 days after aortic constriction in male wild type and *Tlr2* deficient mice

	WT sham	WT TAC	Tlr2^−/−^ sham	Tlr2^−/−^ TAC
TLR1	1.00 ± 0.23	1.69 ± 0.42	1.88 ± 0.34	1.56 ± 0.0.22
TLR4	1.00 ± 0.17	1.36 ± 0.13	0.91 ± 0.05	1.22 ± 0.05
TLR6	1.00 ± 0.03	1.12 ± 0.05	1.03 ± 0.04	1.00 ± 0.07
TLR9	1.00 ± 0.11	0.98 ± 0.01	1.32 ± 0.27	0.92 ± 0.06
CD14	1.00 ± 0.10	1.41 ± 0.18	1.04 ± 0.26	1.22 ± 0.11

Values are normalized to the respective sham group, mean ± SEM, *n* = 3–4/sham group, *n* = 5–8/TAC group

**Table 4:** mRNA and protein expression of pro-inflammatory cytokines 3 days after aortic constriction in male wild type and *Tlr2* deficient mice

	WT sham	WT TAC	Tlr2^−/−^ sham	Tlr2^−/−^ TAC
TNFα	1.00 ± 0.10	1.45 ± 0.15	0.90 ± 0.14	1.21 ± 0.26
IL-1β	1.00 ± 0.28	2.21 ± 0.52	1.19 ± 0.42	1.43 ± 0.21
IL-6	1.00 ± 0.24	5.33 ± 0.97	0.65 ± 0.12	5.74 ± 1.13*
IL-1β protein	1.46 ± 0.25	3.03 ± 0.53	1.53 ± 0.19	2.65 ± 0.44
IL-6 protein	15.84 ± 3.81	19.94 ± 3.68	13.14 ± 2.31	22.42 ± 2.63

mRNA values are normalized to the respective sham group. Protein amount in pg/mg protein. Mean ± SEM, *n* = 3–4/sham group, *n* = 5–8/TAC group

*indicates significant difference to respective sham group (*p* < 0.05, One-way ANOVA with Sidak post-hoc testing)

Next, we analyzed whether hypertrophy related mediators were changed after 3 days of TAC (Table 5). Alterations in TGFβ, ANP and BNP transcription were less prominent than measurements after 14 days of TAC proposed. Interestingly, WT TAC mice but not *Tlr2*^*−/−*^ TAC mice displayed increases of ANP and BNP values with BNP being significantly elevated.

**Table 5:** mRNA expression of prohypertrophic mediators 3 days after onset of aortic constriction in male wild type and *Tlr2* deficient mice

	WT sham	WT TAC	Tlr2^−/−^ sham	Tlr2^−/−^ TAC
TGFβ	1.00 ± 0.04	1.16 ± 0.08	0.93 ± 0.07	1.13 ± 0.05
ANP	1.00 ± 0.27	2.83 ± 0.38	2.16 ± 0.41	3.09 ± 0.49
BNP	1.00 ± 0.19	6.04 ± 1.78^*^	1.82 ± 0.22	4.41 ± 0.49

mRNA values are normalized to wild type sham group. Mean ± SEM, *n* = 3–4/sham group, *n* = 5–8/TAC group. Significant differences (*p* < 0.05, One-way ANOVA with Sidak post-hoc testing) between relevant groups are indicated (*)

## Discussion

Our findings suggest that genetic disruption of *Tlr2* cannot prevent cardiac hypertrophy in a model of hemodynamic overload. On the contrary, *Tlr2* deficiency impaired cardiac hypertrophy after TAC. Increased expression of pro-hypertrophic mediators ANP, BNP and TGFβ after 14 and 28 days of transverse aortic constriction support the finding of enhanced hypertrophy development in *Tlr2* deficient mice.

Since it is unclear which TLRs and danger associated molecular patterns influence cardiac hypertrophy, we examined TLR expressions. We assumed that ligand presentation modulates receptor expression. Interestingly, TLR1 and −4 gene expressions were increased in *Tlr2*^*−/−*^ TAC mice compared to the respective sham group and wild type TAC mice. However, it remains unclear whether up-regulation of TLR1 and −4 was induced by the presence of specific endogenous ligands or was a feedback regulatory event to inflammation. A compensative up-regulation of PRRs in knockout lines might out-balance the respective receptor deficiency, and influence hypertrophy development. However, baseline values of TLRs in cardiac tissue were similar to wild type data. In a previously conducted study our group demonstrated that *Tlr2*^*−/−*^ mice exhibited significantly higher TLR4 baseline levels in aortic tissue, increased pro-inflammatory mediator expression along with a loss of contractile function after 18 h in a Colon ascendens stent peritonitis model [19]. Potentially, increased vascular stress and release of endogenous ligands signaling via TLR4 may have occurred in *Tlr2*^*−/−*^ TAC mice. The TAC induced increase of TLR4 mRNA expression might support the assumption that TLR4 signaling is a major contributor to the development of cardiac hypertrophy. It has already been shown that TLR4 signaling increases early pressure overload dependent cytokine expression [1, 2]. After the detection of elevated TLR4 transcripts after 14 days of TAC, we assumed that TLR4 expression might increase early on and accounts for prolonged inflammation in *TLR2* deficient mice, thereby promoting cardiac hypertrophy development. Weisheit et al. reported that an increased immune cell infiltration and cytokine production was associated with hypertension and end organ damage [20]. Therefore, we analyzed TLR4 and cytokine mRNA and protein levels on day 3 after TAC in wild type and *Tlr2*^*−/−*^ mice. A previous study indicated that aortic constriction in C57BL/6 mice rapidly initiates cytokine induction within 6 h [17], and cytokine levels returned to baseline after 3 days. We did not observe a major load or genotype dependent PRR regulation. Pro-inflammatory cytokine mRNA expression as a measure of the inflammatory response showed a load-dependent elevation in both genotypes with a significant up-regulation of IL-6 mRNA in *Tlr2* deficient mice only. However, protein secretion measured in cardiac tissue was not in line with this observation and serum levels have not been measured. Therefore, the detected elevation of TLR4 and IL-6 mRNA levels are weak indicators of a prolonged inflammatory response in *Tlr2*^*−/−*^ mice. Quantification of immune cells and intracellular cytokine quantification may provide deeper insight into the inflammatory mechanisms involved.

A compensatory left ventricular hypertrophy develops progressively between post-operative days 3 to 10 with minor increase after day 10 [21]. In concordance with these results, we measured a pronounced increase of wild type left ventricular weight in the first 14 days after TAC, without further changes until day 28. Persisting hemodynamic overload induces excessive enlargement of cardiomyocytes and progressive interstitial fibrosis. Furthermore, it results in myocardial microvascular dysfunction, and increased endothelial permeability [22]. Distension of the ventricular wall initiates the secretion of natriuretic peptides, which regulate diuresis and maintenance of blood pressure. In our experiments, TAC caused an increase of natriuretic peptides ANP and BNP as determined on day 14 after surgery. Synthesis of natriuretic peptides is an early load dependent phenomenon starting within 24 h after TAC [23]. ANP and BNP are used as clinical markers for hypertrophy and cardiac dysfunction, which correlate with the severity of symptoms and prognosis [24, 25]. However, it has also been demonstrated that cytokines directly modulate the transcription and translation of natriuretic factors [26]. *Tlr2*^*−/−*^ mice exhibited the strongest increase of ANP and BNP 14 days after TAC, which was in line with enhanced hypertrophy and impaired cardiac function. In contrast, higher ANP and BNP levels were found in wild type mice after 3 days of pressure overload even though differences in the extent of cardiac hypertrophy were not detectable at that time point (data not shown).

Mersmann et al. demonstrated a *Tlr2* deficiency driven adverse cardiac remodeling in a model of myocardial infarction [12]. Twenty-eight days after reperfusion, *Tlr2*^*−/−*^ animals developed left ventricular dilation and defective scar formation. This was associated with pronounced extracellular matrix (ECM) remodeling characterized by reduced collagen and decorin density. In our study, the transcription levels of Col1α1, Col3α1, elastin and LOX were slightly decreased in *Tlr2*^*−/−*^ mice. Thus, an overall lower ECM compound expression in the tissue may favor ECM destabilization. Otherwise, higher cardiac mass in *Tlr2*^*−/−*^ mice may point towards an elevated heart weight due to increased cardiomyocyte mass and size.

Higashikuni et al. concluded from their studies that TLR2 mediated inflammation is essential for adaptive cardiac hypertrophy in response to pressure overload [9]. They also reported that genetic disruption of *Tlr2* impaired hemodynamic function. Furthermore, it enhanced left ventricular dilation and lowered the survival rate. However, in their model *Tlr2* deficiency attenuated cardiac hypertrophy. Even though their and our studies were both performed in the same *Tlr2* knockout mice [27], the extent of hypertrophy, survival rates as well as inflammatory responses differed in numerous aspects, whereas hemodynamic function was to different levels impaired in both studies. Our data demonstrated a compensated hypertrophy with impaired left ventricular diastolic function but preserved contraction and relaxation velocity. In our hands, survival rates of both wild type and *Tlr2*^*−/−*^ mice were above 90 % and did not differ. Even after 28 days of pressure overload, cardiac hypertrophy was still compensated in our study. In contrast, Highashikuni et al. observed a decompensated heart failure alongside with increased mortality within few days in *Tlr2*^*−/−*^ TAC mice*.* A publication elucidating the effect of TAC in commonly used C57BL/6 substrains such as NCrl and J demonstrated that the cardiac response to pressure overload is distinct among the substrains [28]. Backcrossing *Tlr2*^*−/−*^ mice on different BL/6 substrains in various breeding facilities may change the outcome parameters. Furthermore, surgical procedures might vary slightly. For example fabrication by different manufacturers variegates the external diameter of 27G cannulas.

Cardiac hypertrophy displays gender dependent differences. We aimed to elucidate whether gender interferes with the TLR2 dependent development of cardiac hypertrophy. Estrogen and estrogen receptors (ER) play a critical role in cardiac hypertrophy [29, 30]. ERβ signaling protects the murine heart against TAC induced left ventricular hypertrophy [11]. Furthermore, estrogen receptor signaling may impact the responsiveness of TLRs and trigger pro-inflammatory mediator production [31, 32]. An estrogen-response element has been identified in the TLR2 promoter, enhancing TLR2 transcriptional activity in an estrogen dependent pattern [33]. In line with previous reports, we detected an attenuated cardiac hypertrophy of female wild type hearts. However, TLR2 deficiency provoked increased female heart weights after TAC. Future studies need to elucidate whether estrogen-dependent TLR2 transcription occurs upon aortic constriction in female mice, and whether this contributes to the attenuated development of cardiac hypertrophy.

Based on our findings, prospective studies will interrogate the regulation of leukocyte recruitment, activation, and function in models of tissue injury predisposing to secondary infections. Mechanistic analyses need to discover whether a transient modulation of TLR4 signal transduction might offer new possibilities for the better use of safe and efficient TLR4 agonists.

## Conclusions

Our data suggest, that TLR2 signaling may preserve cardiac function and limit cardiac hypertrophy in a murine model of pressure overload. Thus, modulation of TLR2 signaling may provide a future treatment option for cardiac diseases. However, a comprehensive review reveals that the substrain specific phenotype of wild type mice chosen for backcrossing may also influence the extent and pathology of heart failure in *Tlr2* knockout mice. It may alter the expression of TLR2 signaling induced inflammatory mediators as well as the adaption to hemodynamic stress. Therefore, careful regard for mouse strains from different sources is relevant when comparing data and drawing conclusions from independent studies.

## Methods

### Experimental animals

Experiments were performed on male and female mice at an age of about 12 weeks. C57BL/6NCrl mice were purchased from Charles River (Sulzfeld, Germany). Breeding pairs of *Tlr2*^*−/−*^ mice on C57BL/6 genetic background were kindly provided by S. Akira [34]. *Tlr2*^*−/−*^ mice were backcrossed to C57BL/6NCrl. All animals employed in the present study were housed in individually ventilated pathogen-free cages with free access to water and standard rodent chow. The animal protocol was approved by the local committee for animal care (LANUV, Recklinghausen, Germany; animal protocol #50.203.2-BN43 38/06, 9.93.2.10.35.07.157). The protocol was in accordance with the National Institutes of Health guidelines for use of live animals (NIH publication No. 85–23, revised 1996).

### TLR2 genotyping

Genetic modification of each mouse incorporated in the study was confirmed by genotyping. Genomic DNA was extracted from mice tails. The primer sequences used for polymerase chain reaction analysis of the wild type allele were as follows: „TLR2 A“5′-GTT TAG TGC CTG TAT CCA GTC AGT GCG-3′ and „TLR2 B“5′-TTG GAT AAG TCT GAT AGC CTT GCC TCC-3′. „TLR2koCneo“5′-ATC GCC TTC TAT CGC CTT CTT GAC GAC G-3′ and „TLR2 B“were specific for the mutated TLR2 allele.

### Experimental model of transverse aortic constriction

Animals were separated into two subgroups, undergoing TAC or sham operation. TAC induced cardiac hypertrophy in mice. Surgery for TAC was achieved as published previously [17, 35]. Mice were intubated in a supine position and mechanical ventilation was initiated (MiniVent 845, Hugo Sachs Elektronik, March-Hugstetten, Germany). Ventilation was adapted to physiological parameters. A left parasternal incision was performed. Retractors were used to achieve a clear sight into the thorax. A suture was passed underneath the aortic arch and tied down on a 27G needle, which was immediately removed. Thereby, a standardized and previously validated decreased diameter of the aorta was produced [17, 35]. For sham-operation procedure the suture was passed underneath the aortic arch without ligation. After surgery we monitored the mice daily for clinical signs of infection such as shivering, lethargy, and diarrhea. None of the included mice showed any kinds of healing problems following the surgery. For analgesia mice received a single intraperitoneal injection of 0.065 mg/kg BW buprenorphin.

### Hemodynamic measurements

Hemodynamic parameters were recorded at the end of the study period using a 1.2 French pressure catheter (Transonic Systems Inc Ithaca, NY, USA). Animals were prepared under anesthesia with 2.5 Vol.% isoflurane. Data recordings were performed under 1Vol.% isoflurane and 1 L/min oxygen flow. For the recording of left ventricular blood pressure the catheter was inserted into the right carotid artery. First, the catheter was pushed forward to a position 4 mm in front of the aortic valve for peripheral blood pressure recordings and was then further advanced into the left ventricle. Data were analyzed using a power lab data acquisition system (AD Instruments; Software: LabChart for Windows v.6 Power Lab).

### Biometric measurements

The impact on cardiac biometric parameters was investigated 14 or 28 days after TAC or sham surgery. Body weight was registered. Heart and lung were excised, prepared and total heart weight (HW), left ventricular (LVW) as well as lung weights (LW) and tibia lengths (TL) were recorded immediately. Ventricles were snap frozen in liquid nitrogen and kept at −80 °C.

### RNA isolation and quantitative real-time PCR

Total RNA was isolated after homogenization of the left ventricle (TRIzol, Applied Biosystems, Carlsbad, CA, USA). RNA was dissolved in 100 μl of RNase-free water, and concentration was determined photometrically (absorbance at 260 nm) before storage at −80 °C. RNA was transcribed reversely according to the manufacturer’s protocol using the High Capacity cDNA Reverse Transcription Kit (Applied Biosystems, Foster City, CA, USA, Part No. 4368814). 25 μl RNA were mixed with 25 μl master mix, containing 5 μl 10x reverse transcriptase buffer, 2 μl 25x dNTPs, 2 μl 10x random primers, 2.5 μl multi scribe reverse transcriptase and 10.5 μl nuclease free water.

We used specific pre-made TaqMan® Gene Expression Assays (Applied Biosystems) for 18S (Mm02601777_g1), ANP (Mm01255748_g1), BNP (Mm01255770_g1), TGFβ (Mm0044 1726_m1), TNF∝ (Mm00443258_m1), IL-1β (Mm01336189), IL-6 (Mm00446190_m1), CD14 (Mm00438094_g1), TLR1 (Mm01208874_m1), −2 (Mm00442346_m1), −4 (Mm0044 5273_m1), −6 (Mm02529782_s1), −9 (Mm00446193_m1), elastin (Mm00514670), decorin (Mm00514535_m1), lysyl oxidase (LOX)(Mm00495386_m1), collagen 1∝1 (Mm0080 1666_g1), collagen 3∝1 (Mm01254476_m1), collagen 4∝1 (Mm01210125_m1), matrix metalloproteinase (MMP)-2 (Mm00439498_m1), −9 (Mm00442991_m1), and −13. Real-time PCR was performed according to the manufacturer’s protocol. 5.5 ng of cDNA was mixed with 5 μl 2xTaqMan® Universal Master Mix (Applied Biosystems, #4304437), 0.5 μl TaqMan® Gene Expression Assay and 2.3 μl nuclease free water to a final volume of 10 μl in a 384-well optical reaction plate. Each sample was measured in triplicate wells and underwent 40 cycles of amplification on an ABI PRISM® Sequence Detection System (Applied Biosystems). C_T_ values were determined with SDS Software 2.2 (Applied Biosystems) and relative quotients (RQ) were calculated following the ΔΔC_T_ method (RQ target gene / 18S). Fold increase of the wild type sham group was calculated and depicted.

### Protein isolation and enzyme-linked immunosorbent assay (ELISA)

Left ventricular tissue was homogenized in ELISA buffer containing PBS, Igepal (1 μl/ml, Sigma), PMSF (250 mmol in isopropanol, 1 μl/ml, Sigma), and protease inhibitors (Complete mini, Roche). Samples were incubated on ice for 20 min and centrifuged for 15 min at 4 °C and 13,110 g. The supernatant was snap-frozen and used for measuring protein levels with Quantikine mouse tumor necrosis factor (TNF)-α, interleukin (IL)-1β and IL-6 ELISA (R&D Systems, McKinley, MN, USA). The concentration was normalized to protein concentration as determined by BCA protein assay (Pierce).

### Data analysis and statistical procedures

All values are expressed as mean ± SEM. For tests of significance between the groups, one-way analysis of variance (ANOVA) and Tukey or Sidak *post-hoc* testing was performed for statistical analysis. Statistics were calculated using Prism 4.05 (GraphPad Software Inc., San Diego, CA, USA). Differences between experimental groups were considered to be significant with *p* < 0.05.

## Abbreviations

ANOVA: one-way analysis of variance
ANP: atrial natriuretic peptide
BNP: b-type natriuretic peptide
ECM: extracellular matrix
HR: heart rate
HW: heart weight
IL: interleukin
LOX: lysyl oxidase
LVEDP: left ventricular end-diastolic pressure
LVSP: left ventricular systolic pressure
LVW: left ventricular weight
LW: lung weight
MMP: matrix metalloproteinase
TAC: transverse aortic constriction
TGF: transforming growth factor
TL: tibia length
TLR: toll-like receptor
TNF: tumor necrosis factor
WT: wild type (C57BL/6) mice
